# Supplementary material for: Organ transformation by environmental disruption of protein integrity and epigenetic memory in Drosophila
Source: PLoS Biol. 2024 May 28;22(5):e3002629. doi: 10.1371/journal.pbio.3002629 (PMC11161060; doi:10.1371/journal.pbio.3002629)
Supplement: S1 Table — Based on the fraction of pupae and adults presenting bithorax phenocopies. Environmental conditions: Ether, Dechorionation (“Decho”), and no treatment control. Genotypes: yw, trx1+/-, and trx1-/-. Paired Tukey HSD analysis was applied to each of the indicated pairs. (DOCX) [file pbio.3002629.s011.docx]

**Table S1. Significance of gene-environment interaction effect on penetrance**. Based on the fraction of pupae and adults presenting bithorax phenocopies. Environmental conditions: Ether, Dechorionation (‘Decho’), and no treatment control. Genotypes: *yw*, *trx^1+/-^,* and *trx^1-/-^*. Paired Tukey HSD analysis was applied to each of the indicated pairs.

| **Group 1** | **Group 2** | **Adjusted p-value (FDR)** |
| --- | --- | --- |
| *trx^1-/-^*:(-)Ether | *trx^1+/-^*:(-)Ether | 0 |
| *yw*:(-)Ether | *trx^1+/-^*:(-)Ether | 1 |
| *yw* Decho:(-)Ether | *trx^1+/-^*:(-)Ether | 1 |
| *trx^1+/-^*:(-)Ether | *trx^1+/-^*:(+)Ether | 0 |
| *trx^1-/-^*:(+)Ether | *trx^1+/-^*:(-)Ether | 0 |
| *yw*:(+)Ether | *trx^1+/-^*:(-)Ether | 0.0000004 |
| *yw* Decho:(+)Ether | *trx^1+/-^*:(-)Ether | 0 |
| *yw*:(-)Ether | *trx^1-/-^*:(-)Ether | 0 |
| *yw* Decho:(-)Ether | *trx^1-/-^*:(-)Ether | 0 |
| *trx^1+/-^*:(+)Ether | *trx^1-/-^*:(-)Ether | 0.048322 |
| *trx^1-/-^*:(+)Ether | *trx^1-/-^*:(-)Ether | 0 |
| *yw*:(+)Ether | *trx^1-/-^*:(-)Ether | 0.0057505 |
| *yw* Decho:(+)Ether | *trx^1-/-^*:(-)Ether | 0.6117278 |
| *yw* Decho:(-)Ether | *yw*:(-)Ether | 1 |
| *trx^1+/-^*:(+)Ether | *yw*:(-)Ether | 0 |
| *trx^1-/-^*:(+)Ether | *yw*:(-)Ether | 0 |
| *yw*:(+)Ether | *yw*:(-)Ether | 0.0000004 |
| *yw* Decho:(+)Ether | *yw*:(-)Ether | 0 |
| *trx^1+/-^*:(+)Ether | *yw* Decho:(-)Ether | 0 |
| *trx^1-/-^*:(+)Ether | *yw* Decho:(-)Ether | 0 |
| *yw*:(+)Ether | *yw* Decho:(-)Ether | 0.0000004 |
| *yw* Decho:(+)Ether | *yw* Decho:(-)Ether | 0 |
| *trx^1-/-^*:(+)Ether | *trx^1+/-^*:(+)Ether | 0.000002 |
| *yw*:(+)Ether | *trx^1+/-^*:(+)Ether | 0 |
| *yw* Decho:(+)Ether | *trx^1+/-^*:(+)Ether | 0.0000479 |
| *yw*:(+)Ether | *trx^1-/-^*:(+)Ether | 0 |
| *yw* Decho:(+)Ether | *trx^1-/-^*:(+)Ether | 0 |
| *yw* Decho:(+)Ether | *yw*:(+)Ether | 0.0418766 |
