# Supplementary material for: Organ transformation by environmental disruption of protein integrity and epigenetic memory in Drosophila
Source: PLoS Biol. 2024 May 28;22(5):e3002629. doi: 10.1371/journal.pbio.3002629 (PMC11161060; doi:10.1371/journal.pbio.3002629)
Supplement: S2 Table — (DOCX) [file pbio.3002629.s012.docx]

**Table S2. Gene network enrichment analysis of the genes with promoter regions within the highest H3K4me3 retention levels (top 10%)**.

| Gene network | Adjusted p-value |
| --- | --- |
| Spliceosome | 8.58E-32 |
| Ribosome | 2.81E-31 |
| Hippo signaling pathway - fly | 1.35E-28 |
| RNA degradation | 2.96E-24 |
| Ubiquitin mediated proteolysis | 9.23E-24 |
| Endocytosis | 5.66E-23 |
| RNA transport | 2.96E-20 |
| FoxO signaling pathway | 2.96E-20 |
| Wnt signaling pathway | 3.09E-19 |
| Protein processing in endoplasmic reticulum | 2.80E-15 |
| mRNA surveillance pathway | 1.31E-14 |
| Hedgehog signaling pathway | 6.61E-14 |
| TGF-beta signaling pathway | 1.04E-11 |
| mTOR signaling pathway | 2.12E-11 |
| Jak-STAT signaling pathway | 1.87E-08 |
| Notch signaling pathway | 4.31E-08 |
| Phagosome | 0.00348 |
| Regulation of autophagy | 0.0365 |
