# Supplementary material for: Organ transformation by environmental disruption of protein integrity and epigenetic memory in Drosophila
Source: PLoS Biol. 2024 May 28;22(5):e3002629. doi: 10.1371/journal.pbio.3002629 (PMC11161060; doi:10.1371/journal.pbio.3002629)
Supplement: S3 Table — (DOCX) [file pbio.3002629.s013.docx]

**Table S3: ANOVA analysis of the effects of the indicated factors on mean expression of subsets of trx and Ubx targets in haltere discs of 3rd instar larvae**.

| **Set of genes** | **Factor** | **p-value** |
| --- | --- | --- |
| Ubx targets | Genotype | 2.00E-16 |
|  | Ether | 2.68E-11 |
|  | Genotype:Ether | 7.79E-07 |
| trx & Ubx targets | Genotype | 6.34E-15 |
|  | Ether | 1.08E-11 |
|  | Genotype:Ether | 9.95E-05 |
| trx but not Ubx targets | Genotype | 2.28E-08 |
|  | Ether | 1.33E-08 |
|  | Genotype:Ether | 3.64E-05 |

.
