# Supplementary material for: Organ transformation by environmental disruption of protein integrity and epigenetic memory in Drosophila
Source: PLoS Biol. 2024 May 28;22(5):e3002629. doi: 10.1371/journal.pbio.3002629 (PMC11161060; doi:10.1371/journal.pbio.3002629)
Supplement: S4 Table — (DOCX) [file pbio.3002629.s014.docx]

**Table S4: ANOVA analysis of the effects of the indicated factors on mean expression of wing-related targets of Ubx in haltere discs of 3rd instar larvae**.

| Set of genes | Factor | p-value |
| --- | --- | --- |
| Wing-related genes | Genotype | 8.91E-16 |
|  | Ether | 1.51E-09 |
|  | Genotype:Ether | 8.51E-07 |
| Wing-related genes (also Ubx targets) | Genotype | 8.62E-16 |
|  | Ether | 1.28E-09 |
|  | Genotype:Ether | 1.57E-06 |
