# Supplementary material for: Organ transformation by environmental disruption of protein integrity and epigenetic memory in Drosophila
Source: PLoS Biol. 2024 May 28;22(5):e3002629. doi: 10.1371/journal.pbio.3002629 (PMC11161060; doi:10.1371/journal.pbio.3002629)
Supplement: S5 Table — (DOCX) [file pbio.3002629.s015.docx]

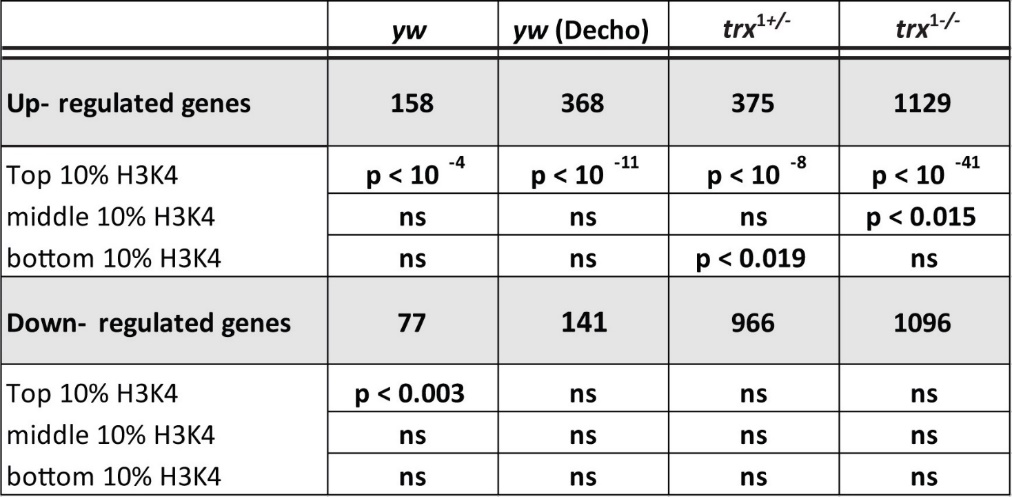
**Table S5:** Significance of overlap between H3K4me3 levels in exposed embryos (4.5hr AED) and differential expression (ether vs. control) in the haltere discs of 3rd instar larvae. Shown for *yw* (with and without dechorionation), *trx*^1-/+^, and *trx*^1-/-^. Hypergeometric te
