# Supplementary material for: Organ transformation by environmental disruption of protein integrity and epigenetic memory in Drosophila
Source: PLoS Biol. 2024 May 28;22(5):e3002629. doi: 10.1371/journal.pbio.3002629 (PMC11161060; doi:10.1371/journal.pbio.3002629)
Supplement: S6 Table — (DOCX) [file pbio.3002629.s016.docx]

**Table S6: Primer sequences for qPCR analysis** **of mRNA levels of wing genes**

| **Target gene** | **Forward primer** | **Reverse primer** |
| --- | --- | --- |
| *act5C* | CCCTCGTTCTTGGGAATGG | CGGTGTTGGCATACAGATCCT |
| *wg* | CCAAGTCGAGGGCAAACAGAA | TGGATCGCTGGGTCCATGTA |
| *bs* | CGCTACACGACCTTCTCCAA | AGTTGAGGCAGGTCTGGATG |
| *vg* | GGGGCAATACCCAAGAATCTG | TTGGTGAACACAACGCAGGA |
| *ac* | GCTTTGGGCAGCGAAAATCAC | GCTTTGGGCAGCGAAAATCAC |
| *salr* | CCGGAGAACAGTAACGAGGC | GCCACTTTGGTATGTTGTAGAGC |
| *ash2* | AGCCACACACTCTGTGAAC | GTCGGGCATCTCTTCGATGG |
| *exd* | ATGATGGCTCCGCAAGGATAC | GTCAAGTGATTGCTCCGAGATAC |
| *hth* | CACCACTCTCCGCACATGAC | TCGGGTATTGCGCCCATTAG |
| *ast* | GCCAGGAAACTTAACTGCCCA | ATCAGCGGGATGTACTTGACG |
