# Supplementary figures and images for: Organ transformation by environmental disruption of protein integrity and epigenetic memory in Drosophila
Source: PLoS Biol. 2024 May 28;22(5):e3002629. doi: 10.1371/journal.pbio.3002629 (PMC11161060; doi:10.1371/journal.pbio.3002629)

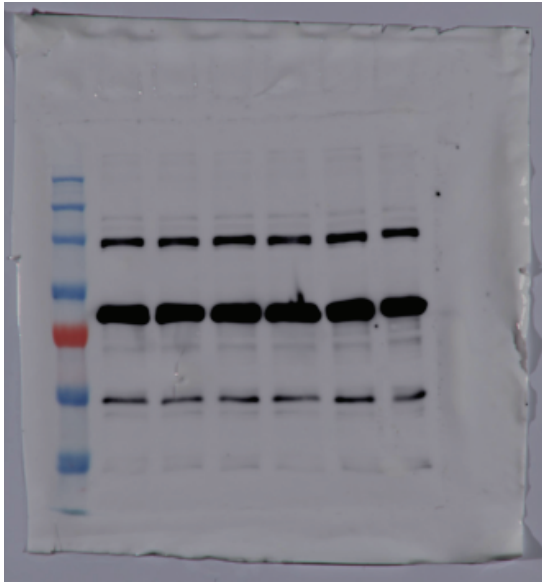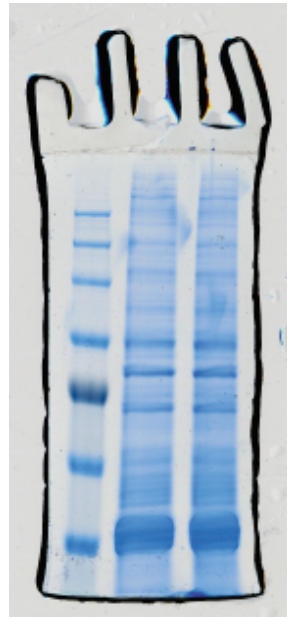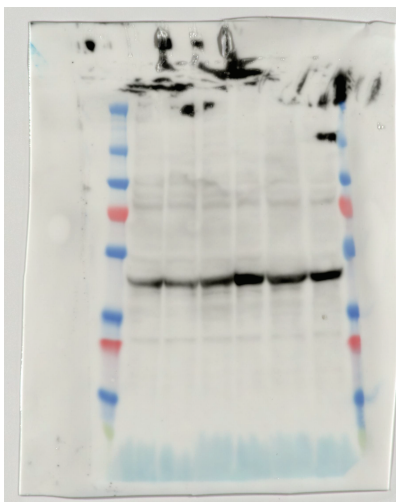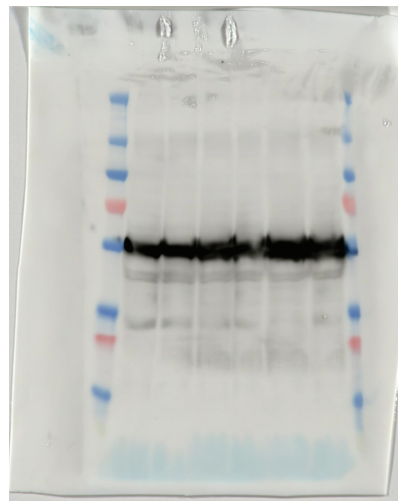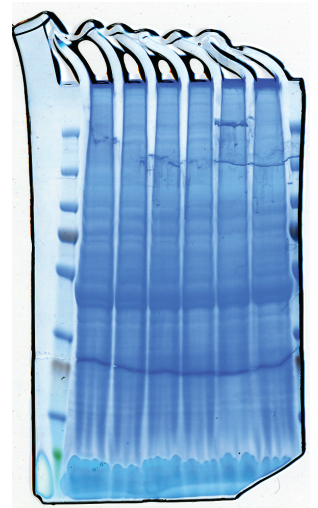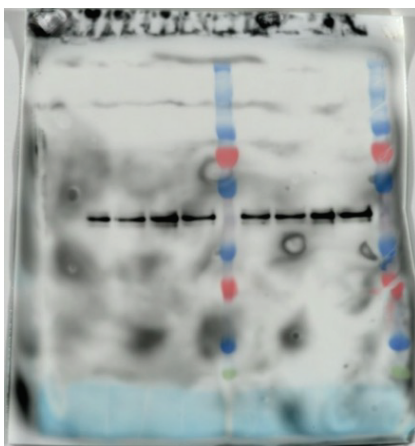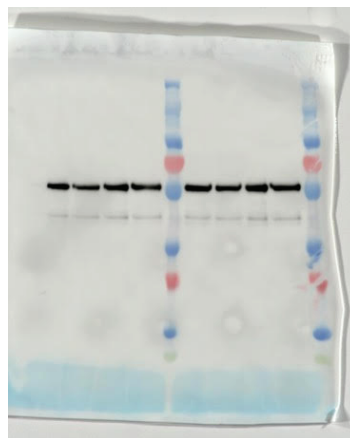

Supplement: S1 Raw Images — (PDF) [file pbio.3002629.s025.pdf]
