# Supplementary material for: Organ transformation by environmental disruption of protein integrity and epigenetic memory in Drosophila
Source: PLoS Biol. 2024 May 28;22(5):e3002629. doi: 10.1371/journal.pbio.3002629 (PMC11161060; doi:10.1371/journal.pbio.3002629)
Supplement: Attachment — Submitted filename: Response to reviewers.docx [file pbio.3002629.s026.docx]

**Point-by-point responses to the reviewer’s comments**

**Reviewer 1**

***Minor***

***Comment 1:*** *“RNA-seq performed 1 hour after ether exposure of early embryos did not reveal a strong transcriptional response. This was interpreted to mean that H3K4me3 predisposes genes for a transcriptional response later in development. However, the authors should consider the likely possibility that 1 hour is insufficiently long to see a shift in steady-state mRNA levels (measuring changes in nascent transcription may have revealed a stronger transcriptional response)”*

**Response:**

We have performed additional experiments to address the reviewer’s suggestion by qPCR analysis of the mRNA levels of key wing genes 4 hours after ether exposure vs. control. This analysis was applied to genes that are known to be involved in wing formation and are also up-regulated in the haltere discs of larvae that are developed from ether-exposed embryos. This analysis confirmed the lack of change in the embryonic mRNA levels of wing genes (New Supplementary Fig. S4E). We applied this analysis to a panel of key wing genes that are known to be involved in wing formation and are also upregulated in the haltere disc of larvae that are developed from ether-exposed embryos.

***Comment 2:*** *“I believe that the order in which the figures and authors' conclusions are presented could be improved for greater clarity. See the overview in which I propose a possibly more intuitive flow of arguments”*

**Response:**

We adopted this recommendation and revised the flow of the description as advised by the reviewer. We thank the reviewer for this helpful suggestion.

***Comment 3:*** *“Are Trx protein levels reduced upon genetic or chemical perturbation of Hsp90? Showing this could strengthen the proposed relationship of Trx as a substrate of Hsp90”*

**Response:**

We were hoping to address this with Western blot measurements of the Trx protein levels (as we did for Ubx and Hsp90), but we could not find a suitable antibody against Trx that would have allowed us to address this question within a reasonable time frame for revision. We therefore rely on the demonstration of Tariq et al. (2009) of reduced Trx levels by chemical inhibition of Hsp90.

***Comment 4:*** *“Ether suppresses H3K4me3 trimethylation" should be "H3K4 trimethylation”*

**Response:**

Changed as advised.

***Comment 5:*** *“Fig. 1A: please indicate the time point for the proteomics experiment. I believe this information was also missing from the Methods”*

**Response:**

The time point is now indicated in Fig. 1A and the *Methods* section.

***Comment 6:*** *“Fig. 1: please show a couple of screenshots of K4me3 profiles at example loci, to complement the global analyses presented in this figure”*

**Response:**

We added the recommended screenshots of H3K4me3 (as well as H3K27me3) profiles in a new supplementary figure (Fig. S3).

***Comment 7:*** *“Fig. 1J: please clarify whether the plotted data corresponds to the ether-treated or control conditions”*

**Response:**

The original Fig. 1J displayed the H3HK4me3 distributions (of all genes and wing genes) in ether-treated embryos. The distributions for control embryos were left out to reduce the complexity of the figure. However, following this comment (as well as comment 10 of reviewer 3), we added the respective distributions of non-exposed embryos (revised Fig. 3F).

***Comment 8:*** *“Fig. 2A: please specify in the figure legend or main text how Trx protein levels were quantified”*

**Response:**

A description of the quantification of the relative levels of Trx protein in ether-exposed vs. control embryos was added to the legend as advised (revised Fig. 1J).

***Comment 9:*** *“Fig. 2F: please* *swap the order of the microscopy images, placing the Ctrl on top”*

**Response:**

The order of images was swapped as advised (revised Fig. 1I).

***Comment 10:*** *“Fig. 2: please clarify what happens to protein levels of Ubx, in addition to quantifying Trx protein levels (in Fig. 2A). This remains an important point to clarify - is upregulation of wing genes in halteres of animals exposed to ether early in development caused by decreased Ubx protein levels or to* *an inability of Ubx to repress its target genes?”*

**Response:**

We agree with the importance of clarifying this point. Accordingly, we performed additional experiments to determine what happens to the protein levels of Ubx and whether the upregulation of wing genes may also be explained by decreased Ubx levels. In the revised manuscript, we added quantitative western blot measurements of Ubx protein levels in the embryo as well as in the haltere disks of 3^rd^ instar larva. In either case, we found that exposure to ether does not reduce the protein levels of Ubx, indicating that the induction of wing genes in the haltere is not caused by downregulation of Ubx (a known suppressor of these genes). These findings are provided in new figures (Fig. 1L and Fig. S2 for the embryo data and Fig. S7 for the haltere). Moreover, the transcriptomics analysis of *yw* and *trx*^1^ embryos shows that ether does not upregulate the Ubx targets in the embryo (p = 0.15 for *yw* and p = 0.19 for *trx*^1^, Hypergeometric test), providing additional evidence that Ubx function is unaffected by the exposure.

***Comment 11:*** *“The meaning of "reaction" was unclear in the sentence: "it suggests that the increase in bithorax reaction in embryos with reduced Hsp90...”*

**Response:**

We revised this sentence and revised the manuscript for clarity. The modified sentence is: “these findings suggest that loss-of-function of *Hsp90* aggravates the bithorax induction by contributing to the reduction of *trx* function”.

***Comment 12:*** *“Fig. 3D: from the x axis label it is unclear that the last column corresponds to double heterozygotes”*

**Response:**

We changed the label to specifically indicate that this column corresponds to double heterozygotes (*Hsp83*^e6A-/+^/*trx*^1-/+^).

***Comment 13:*** *“There is an incomplete sentence in the first paragraph of the section "Ether exposure up-regulates wing-related Ubx-trx targets in the haltere”*

**Response:**

The sentence was corrected.

***Comment 14:*** *“Upregulation of wing genes in the haltere correlates with H3K4me3 levels at the time of exposure": "shortly after exposure" would be more accurate than "at the time of exposure" because it is assessed after exposure, not before. "At the time of exposure" is also repeated in the Discussion”*

**Response:**

We adopted the reviewer’s suggestion and revised the text accordingly.

***Comment 15:*** *“There are several wrong references to figure panels, as in the couple of examples listed below: a. Fig. 2 legend: references to figure panels are mixed up. b. The reference to Fig. 6A in the Supplementary discussion is wrong”*

**Response:**

We corrected the references. We thank the reviewer for pointing this out.

***Comment 16:*** *“Please specify how many biological replicates were generated for all genome- or proteome-wide datasets”*

**Response:**

We used 3 biological replicates for the proteomics and RNA-seq analyses, while the ChIP-seq analysis was performed with 2 biological replicates (standard practice). This is now specified in the corresponding legends of Figs. 1 and 3.

**Reviewer 2**

***Minor***

***Comment 1:*** *“The Introduction is extremely short, especially for a study that is revisiting historical observations. Introduction of the historical mystery (i.e. the "Waddington Revisited" portion of the manuscript title), is only briefly mentioned in the first paragraph of the Introduction. The first several paragraphs of the Discussion would be more helpful in the Introduction, as well as more attention paid to the original Waddington discovery and any subsequent follow up (or lack thereof)”*

**Response:**

We extended the introduction in accordance with the reviewer’s suggestion and the editorial request to explain the importance of this investigation beyond its historical significance. The revised introduction highlights outstanding open questions regarding the mechanistic basis of environmental induction of morphological transformations and explains why this is important for understanding the evolutionary process and potentially also for developing treatments to suppress (and/or reverse) harmful malformations.

***Comment 2:*** *“In the first section of the Results, the authors jump from phenotypic observations to ChIP-seq for H3K4me3 and H3K27me3, without explaining why they chose these marks. It is apparent later, with the introduction of trx, but not at this point in the Results section. This logic leap was very distracting when reading the manuscript. Can the authors fill in the logic here?”*

**Response:**

We agree that the original description was rather jumpy and we modified the flow line to avoid distracting logic leaps (as well as “narrative jumps” that are mentioned in comment 3).

***Comment 3:*** *“The narrative jumps around, forming the model in steps that are not linear. From protein misfolding to epigenetic misregulation, then back to protein misfolding and Hsp90. It's not clear in this narrative (until the model) that Hsp90 is operating upstream of trx. The authors can make this clearer in the narrative by building the model sequentially with the figures and introducing the histone modifications later”*

**Response:**

We adopted the reviewer’s recommendation and revised the narrative accordingly.

***Comment 4:*** *“Fig 1: B - The halter is difficult to see. We suggest a blow-up image of this region”*

**Response:**

We replaced the image and added a blow-up as advised (revised Fig. 1B).

***Comment 5:*** *“Fig 1: I - Figure legend lists red for up-regulated GO terms, rather than yellow”*

**Response:**

The specified color was changed to “orange” (now in revised Fig. 3E).

***Comment 6:*** *“Fig 1: K- Some problems with the text”*

**Response:**

The text problem has been fixed (now in revised Fig. 3G).

***Comment 7:*** *“Fig 1: L - Define CDF (y axis)”*

**Response:**

CDF (Cumulative density function) is now defined in the legend of the revised Fig. 3H.

***Comment 8:*** *“Fig 2: A - Relative to what?”*

**Response:**

The protein levels are relative to the average level in untreated control. We changed the y-axis to “Rel. protein level” and revised the legend for clarity (revised Fig. 1J).

***Comment 9:*** *“Fig 2: B - Something is wrong with the legend, as B is not accurately represented”*

**Response:**

The legend was corrected (now in revised Fig. 1E).

***Comment 10:*** *“Fig 2: D - Define MDEG*ML/MG”*

**Response:**

We changed the y-axis to “mDeg*ml/mg” (millidegrees*milliliter/milligram) as specified in the legend (revised Fig. 1G).

***Comment 11:*** *“Fig 2: G - Keep nomenclature consistent and replace hsp83 with Hsp90. Statistics required?”*

**Response:**

We changed the nomenclature for consistency and specified the statistics in the legend (now in revised Fig. 1K).

***Comment 12:*** *“Fig 2:H - In legend "Same as (F)" should read "same as (G)”*

**Response:**

The legend was changed (now in revised Fig. 1M).

***Comment 13:*** *“Fig 3: A -The haltere is too small to see”*

**Response:**

We replaced the image and incorporated a large blow-up enabling inspection of details (revised Fig. 2A).

***Comment 14:*** *“Fig 3:A-D are all similar data. Why are B and C presented as boxplots, while A and D are presented as bar graphs? Consider making all four of these graphs consistent in data presentation and labeling, for clarity”*

**Response:**

We adopted the reviewer’s suggestion (now in the revised Fig. 2).

***Comment 15:*** *“Fig 3: D - What does "Average no." mean? Out of how many total?”*

**Response:**

We changed the display to indicate the percentage of *Hsp83*^e6A-/+^/*trx*^1-/+^, *trx*^1-/-^ and *Hsp83*^e6A+/-^ flies exhibiting bithorax phenocopies. The revised legend now indicates that this box plot represents 4 biological replicates, each with approximately 100 flies (revised Figure 2D).

***Comment 16:*** *“An unfinished sentence: Analysis of mRNA levels of trx, Ubx and their gene targets ()”*

**Response:**

We revised the text and thank the reviewer for pointing this out.

***Comment 17:*** *“Fig 4:B - This should not be a regression. There should not be connection between the genotypes; they are discrete”*

**Response:**

We removed the dotted line to avoid wrong impression of connection between genotypes.

***Comment 18:*** *“Fig 5:A - How is this study controlled for total mRNA? Again, this should not be a linear graph, as the Y axis is discrete, rather than continuous”*

**Response:**

Fig. 5A compares average mRNA levels of distinct gene subsets of the same sample (the subsets are defined by high and low H3K4me3 levels). Since the total RNA level in the sample is the same for both subsets, the average mRNA levels of the two gene subsets can be directly compared without a need to control for total RNA. For each sample, we computed the average FPKM (standard mRNA normalization) for two distinct subsets of genes, exhibiting, respectively, high and low H3K4me3 levels. The y-axis displays the average FPKM for each subset (with and without ether exposure) based on 3 independent replicates.

The reviewer is, of course, correct about the discreteness of the graph and we removed lines to avoid potential confusions.

***Comment 19:*** *“Fig 6: A - this model is very clear. B - "Survival" should be "Survival* *when exposed to ether vapor. Missing word in legend "during most OF the syncytial period”*

**Response:**

We are pleased that the model was found to be clear and we incorporated the suggested text changes in the legend of panel B.

***Comment 20:*** *“The first paragraph of the Supplementary Discussion is important, and should be included in the main text, if possible. The other paragraphs of this section are largely redundant with what the authors have already stated or the model figure”*

**Response:**

We adopted this suggestion and incorporated the first paragraph of the Supplementary Discussion in the main text.

**Reviewer 3**

***Major***

***Comment 1:*** *“The authors seem to assume that changes in H3K4me3 levels are responsible for gene expression changes, possibly through altered Ubx levels. Unfortunately, while H3K4me3 levels are correlated with active transcription, there is not a lot of data to support H3K4me3 directly influences transcription. In fact, in Drosophila, H3K4me3 does not significantly affect transcription (Hodl and Baller 2012 Current Biology). This is part of larger issue I have with much of the manuscript that the authors provide a lot of correlative data between transcription and H3K4me3, which would be expected given the relationship between those two things. For example, what if the proteotoxic stress just reduces Ubx levels and/or activity? You would get the same phenotype completely independent of Trx and H3K4me3. However, combining trx and hsp83 mutations would also give you increased bithorax phenocopy, but for a slightly different reason”*

**Response:**

We performed additional experiments to investigate the possibility that the bithorax phenocopies are caused by a reduction in Ubx levels. The new Western blot analysis of Ubx protein levels in ether-exposed and non-exposed embryos showed that Ubx is not repressed by the proteotoxic stress (new Fig, 1L; new Supplementary Fig. S2). Moreover, analysis of the haltere disc of 3rd instar larvae also revealed no change in the Ubx protein level (new Supplementary Fig. S7), indicating that the upregulation of *Ubx* targets in the haltere disc (revised Fig. 4) is not caused by down-regulation of Ubx. Analysis of the transcriptomics data from *yw* and *trx*^1^ embryos further showed that ether does not upregulate the Ubx targets in the embryo (Hypergeometric test, p = 0.15 for *yw* and p = 0.19 for *trx*^1^), providing additional evidence that ether does not affect Ubx functionality.

In agreement with the reviewer’s comment, we showed in the original submission that the changes in H3K4me3 do not affect the transcription in the embryo (now in Fig. 3D, E; Supplementary Fig. S4B-E). We do show, however, that Ubx targets (wing genes) that are later upregulated in the larval haltere disc retain higher levels of H3K4me3 following the exposure. Taken together with the reported contribution of H3K4me3 to the stabilization of active transcription (Hörmanseder et al., 2017; Pinney et al., 2011), these findings suggest that the differential retention of H3K4me3 in wing genes loci opposes their normal silencing in the haltere. This is further supported by the aggravated response on the background of additional loss of *trx* function.

***Comment 2:*** *“There is not enough information to evaluate the reproducibility of the mass spec data. This type of analysis is complicated without using quantitative mass spec methods or some type of spike in control. The methods don't even indicate how many replicates were performed for each condition. To begin to even assess this data, readers would need much more information. Additionally, in Figure 2H is hard to interpret. The authors are claiming in the text that there are higher protein levels of Hsp90&Ubx&Trx targets. But, they present a 'fold change' of less than 1, indicating that these protein levels are reduced. This could be a misinterpretation of the figure, but there is not information in the legend to properly interpret this data”*

**Response:**

We added detailed information about the mass spec analysis (both in the methods and in revised Fig. 1J, K, L-N). The mass spectrometry analysis is based on 3 replicates of each condition. Each sample was processed according to standard procedures, and the protein levels were normalized using label-free quantification method (Cox et al., 2014).

The original Fig. 2H displayed changes in protein levels using a non-suitable measure and we would like to thank the reviewer for noticing this problem. The corrected analysis (and legend) are now provided in the revised Fig. 1M. It displays the median protein fold change for the following subsets of Hsp90 clients: All Hsp90 clients (n=280), Hsp90 w/o Trx or Ubx targets (n=197), Hsp90 & Trx but not Ubx targets (n=52), Hsp90 & Trx targets (n=69), Hsp90 & Ubx targets (n=31), Hsp90 & Trx & Ubx targets (n=16). The revised figure also includes p-values (*a*, *b*, *c*, *d*) indicating that the protein fold-change of Hsp90 clients that are also targets of Trx & Ubx is significantly higher than in all the subsets of Hsp90 clients that do not include the Ubx targets.

***Comment 3:*** *“Why does the penetrance in 3A not align with the penetrance in 1C? Wild type should give ~0.8 with ether based on 1C”*

**Response:**

The reason for the apparent difference between the original Fig. 1C and Fig. 3A is two-fold:

1) Fig. 3A displayed the mean penetrance, while the horizontal bar in the box plot of Fig. 1C corresponds to the median penetrance. Since we agree that the use of different displays can be confusing, the revised manuscript presents these data sets using the same type of box plot (as advised in comment 14 of reviewer 2).

2) The original figures are based on distinct sets of penetrance measurements. Since the penetrance in the original Fig. 3A is compared across different conditions, the wild-type results in this experiment must be kept separate from the result of the experiment that produced Fig. 1C. Note, though, that the result in Fig. 3A is clearly within the deviation range of Fig. 1C.

***Comment 4:*** *“I still have a hard time understanding the logic of the proposed model. Proteotoxic stress caused a reduction in Trx protein and subsequent H3K4me3 levels. But many wing-related genes have high levels of H3K4me3 and are somehow not affected by dampened Trx activity. How do you explain this?”*

**Response:**

Perhaps we should have explained this more clearly in the original submission. Wing-related genes are affected by dampened Trx activity. Despite this dampening, wing-related genes still exhibit significantly higher levels of H3K4me3 post-exposure compared to other genes (new Fig. 4F, supplementary Fig. S4F). To explain this, we examined the following parameters:

(1) **H3K4me3 absolute Levels:** Wing-related genes have initially higher H3K4me3 levels compared to other genes, as can be seen in the non-exposed embryos (new Fig. 4F; supplementary Fig. S4F, G; (Li et al., 2014)).

(2) **H3K4me3 Retention**: In the original submission, we showed that the degree of dampening of H3K4me3 marks (defined as the ratio of H3K4me3 levels in exposed to non-exposed) correlates with the transcriptional activity at the respective locus (Fig. 4H; supplementary Fig. S5A-D).

These findings suggest that the initially higher H3K4me3 levels at wing genes and the preferential retention of H3K4me3 due to active transcription of wing genes at the time of exposure account for the higher post-exposure levels of H3K4me3 at wing genes vs. bulk.

***Comment 5:*** *“I found much of the transcriptional analysis in Figures 5 to be redundant and at times hard to follow. It might be helpful to put some of this data into the Supplement and focus on the really key data. In the same vein, I had a good grasp on the model proposed by the authors, but Figure 6 was hard to follow”*

**Response:**

In accordance with the reviewer’s suggestion, we moved the original Fig. 5B to supplementary data (revised Supplementary Table S5) and revised Fig. 6 for clarity.

***Minor***

***Comment 6:*** *“Fig 1C: should have a better label on Y axis. Shouldn't have to go to the legend to know what is being plotted”*

**Response:**

The label was changed to “% Bithorax phenocopy” for clarity.

***Comment 7:*** *“Text issues in the label on 1I, 1K - could be form the PDF conversion?”*

**Response:**

We believe it was indeed due to conversion issues. We corrected the text is now corrected (revised Figs. 3E and 3G) and we thank the reviewer for noticing this issue.

***Comment 8:*** *“Additionally, it would be nice to know what targets of Trx and Ubx were used to generate the data in 2H”*

**Response:** The targets of Trx and Ubx that were used to generate the data are given by the intersection of the Hsp90 client specified in (Hartson & Matts, 2012) with the set of Trx targets (Enderle et al., 2011) and/or Ubx targets (Consortium et al., 2010; Nègre et al., 2011).

***Comment 9:*** *“What is PDF on 1J? Some measure of density? Also, is this promoter specific, throughout the gene body, or both?”*

**Response:** “PDF” in the original Fig. 1J corresponds to “Probability Density Function”, as now indicated in the legend (revised Fig. 3F). The data is based on gene-specific measurements of H3K4me3 levels in both the promoter and coding regions of the gene.

***Comment 10:*** *“How do the wing specific genes change in response to ether? Same as bulk or are they less affected?”*

**Response:**

In the revised manuscript, we added the respective distributions of H3K4me3 in non-exposed embryos, demonstrating that: (1) the H3K4 tri-methylation in the bulk is shifted toward lower levels in ether-exposed embryos (solid vs. dotted gray line), (2) the H3K4 tri-methylation in wing genes is also repressed by ether (solid vs. dotted red line), but (3) retain significantly higher levels compared to the bulk (solid red vs. dotted red line). This data is now included in the revised Fig. 3F.

***Comment 11:*** *“trx(protein)>TRX or Trx”*

**Response:**

We changed to “Trx” as advised.

***Comment 12:*** *“drosophila > Drosophila - throughout manuscript”*

**Response:**

Changed to “Drosophila” as advised.

***Comment 13:*** *“excess reduction of trx function > reduction of Trx function (the excess makes it very confusing)”*

**Response:**

We made the recommended change.

**References**

Consortium, T. modENCODE, Roy, S., Ernst, J., Kharchenko, P. V, Kheradpour, P., Negre, N., Eaton, M. L., Landolin, J. M., Bristow, C. A., Ma, L., Lin, M. F., Washietl, S., Arshinoff, B. I., Ay, F., Meyer, P. E., Robine, N., Washington, N. L., Di Stefano, L., Berezikov, E., … Zieba, J. (2010). Identification of Functional Elements and Regulatory Circuits by *Drosophila* modENCODE. *Science*, *330*(6012), 1787–1797. https://doi.org/10.1126/science.1198374

Cox, J., Hein, M. Y., Luber, C. A., Paron, I., Nagaraj, N., & Mann, M. (2014). Accurate Proteome-wide Label-free Quantification by Delayed Normalization and Maximal Peptide Ratio Extraction, Termed MaxLFQ. *Molecular & Cellular Proteomics*, *13*(9), 2513–2526. https://doi.org/10.1074/mcp.M113.031591

Enderle, D., Beisel, C., Stadler, M. B., Gerstung, M., Athri, P., & Paro, R. (2011). Polycomb preferentially targets stalled promoters of coding and noncoding transcripts. *Genome Research*, *21*(2), 216–226. https://doi.org/10.1101/GR.114348.110

Hartson, S. D., & Matts, R. L. (2012). Approaches for defining the Hsp90-dependent proteome. *Biochimica et Biophysica Acta (BBA)-Molecular Cell Research*, *1823*(3), 656–667. https://doi.org/10.1016/j.bbamcr.2011.08.013

Hörmanseder, E., Simeone, A., Allen, G. E., Bradshaw, C. R., Figlmüller, M., Gurdon, J., & Jullien, J. (2017). H3K4 methylation-dependent memory of somatic cell identity inhibits reprogramming and development of nuclear transfer embryos. *Cell Stem Cell*, *21*(1), 135–143. https://doi.org/https://doi.org/10.1016/j.stem.2017.03.003

Li, X.-Y., Harrison, M. M., Villalta, J. E., Kaplan, T., & Eisen, M. B. (2014). Establishment of regions of genomic activity during the *Drosophila* maternal to zygotic transition. *Elife*, *3*, e03737. https://doi.org/https://doi.org/10.7554/eLife.03737

Nègre, N., Brown, C. D., Ma, L., Bristow, C. A., Miller, S. W., Wagner, U., Kheradpour, P., Eaton, M. L., Loriaux, P., Sealfon, R., Li, Z., Ishii, H., Spokony, R. F., Chen, J., Hwang, L., Cheng, C., Auburn, R. P., Davis, M. B., Domanus, M., … White, K. P. (2011). A cis-regulatory map of the *Drosophila* genome. *Nature*. https://doi.org/10.1038/nature09990

Pinney, S. E., Jaeckle Santos, L. J., Han, Y., Stoffers, D. A., & Simmons, R. A. (2011). Exendin-4 increases histone acetylase activity and reverses epigenetic modifications that silence Pdx1 in the intrauterine growth retarded rat. *Diabetologia*, *54*, 2606–2614. https://doi.org/https://doi.org/10.1007/s00125-011-2250-1

Tariq, M., Nussbaumer, U., Chen, Y., Beisel, C., & Paro, R. (2009). Trithorax requires Hsp90 for maintenance of active chromatin at sites of gene expression. *Proceedings of the National Academy of Sciences*, *106*(4), 1157–1162. https://doi.org/https://doi.org/10.1073/pnas.0809669106
